# Asymmetries Between Direct and Indirect Scalar Implicatures in Second Language Acquisition

**DOI:** 10.3389/fpsyg.2019.00877

**Published:** 2019-04-24

**Authors:** Shuo Feng, Jacee Cho

**Affiliations:** Department of English, University of Wisconsin-Madison, Madison, WI, United States

**Keywords:** direct and indirect scalar implicatures, alternatives, SI suspension, second language acquisition, covered-box paradigm

## Abstract

A direct scalar implicature (DSI) arises when a sentence with a weaker term like *sometimes* implies the negation of the stronger alternative *always* (e.g., John sometimes (∼ not always) drinks coffee). A reverse implicature, often referred to as indirect scalar implicature (ISI), arises when the stronger term is under negation and implicates the weaker alternative (e.g., John doesn’t always (∼ sometimes) drink coffee). Recent research suggests that English-speaking adults and children behave differently in interpreting these two types of SI (Cremers and Chemla, 2014; Bill et al., 2016). However, little attention has been paid to how these two types of SI are processed in a non-native, or second language (L2). By using a covered box paradigm, this study examines how these two types of SI are computed and suspended in a second language by measuring the visible vs. covered picture selection percentage as well as response times (RTs) taken for the selection. Data collected from 26 native speakers of English to 24 L1-Chinese L2-English learners showed that unlike native speakers, L2 speakers showed asymmetries in their generation and suspension of DSI and ISI. That is, L2 speakers computed DSI more often than ISI, but they suspended ISI more frequently than DSI. Furthermore, our RT data suggested that L2 speakers suspended ISI not only more frequently but also significantly faster than DSI. Regarding the asymmetrical behavior among L2 speakers, we consider the number of alternative meanings involved in DSI vs. ISI suspension and different routes to the suspension of SI.

## Introduction

Many linguistic forms are interpreted semantically and pragmatically, which generate more than one meaning from the same form. This forces the hearer to consider all the alternative meanings and choose the meaning that is most appropriate in a given context. Alternative meanings are argued to be accessed and computed separately from the semantic meaning (Rooth, 1985, 1992, 2016). For example, the utterance in (1a) has the semantics of (1b) but implicates the proposition in (1c). Similarly, (2a) can be interpreted semantically as in (2b) and also pragmatically as in (2c). (The symbol “∼” in this paper is used to indicate implied meaning).

(1) a. Bob sometimes went to school (DSI).
  b. Bob went to school at least once and possibly all the time (always).
  c. ∼ Bob didn’t always go to school.

(2) a. Bob did not always go to school (ISI).
  b. Bob failed to go to school at least once and possibly never went to school.
  c. ∼ Bob sometimes went to school.

The linguistic phenomenon that involves a set of alternatives in terms of informational strength (e.g., < *never, rarely, sometimes, often, always >, < some, most, all* >) is called scalar implicature (SI). Generating an implicature from a weaker term like *sometimes* by negating the stronger alternative *always*, as in (1a) and (1c), is often referred to as *direct scalar implicature* (DSI). An implicature derived from the stronger term under negation by considering the weaker alternative like (2a) and (2c) is called *indirect scalar implicature* (ISI).

An account for *why* and *how* we make inferences like (1c) and (2c) beyond what was said in (1a) and (2a) comes from the philosopher Grice’s (1975) theory of inferential communication. According to the theory, we conduct our communication based on rational expectations and principles to meet the goals of communication. He called these principles and expectations ‘maxims’. One of the maxims, the *Quantity Maxim*, states that interlocutors are cooperative by making their contribution as informative as is required but no more informative than is required. On this account, saying *Bob sometimes went to school* while he always went to school is true but underinformative, thus violating the Quantity Maxim. This prompts the hearer to make the inference that the stronger term *always* does not hold since the speaker would have said *Bob always went to school* following the Quantity Maxim.

Drawing on Grice’s theory of inferential communication, Levinson (2000) proposes a Default Inference account of scalar implicatures to explain how scalar inference arises in real-time communications. According to Levinson (2000), scalar implicatures are default inferences that are generated automatically and are canceled only when the context calls for it. Scale terms such as *sometimes* are stored in our memory in association with alternative terms like *always*, *often*, and *rarely* due to habitual generation of the implicatures for *sometimes* (i.e., ‘not always’) in everyday communications (Gazdar, 1979; Levinson, 1983, 2000). Since scalar implicatures are made by default, they require little cognitive efforts from a processing point of view. Some recent psycholinguistic studies on adult native speakers provided evidence for the Default account (Grodner et al., 2010; Lewis and Phillips, 2011).

Arguing against the default view is a context-driven view such as the Relevance Theory supported by Sperber and Wilson (1986/1995) and Carston (2004). Within this approach, utterances are enriched with inferences only if they are relevant to reach the speaker’s intended meaning in a given context. From the point of view of the Relevance Theory, the implicated meaning of *sometimes* (∼ *not always*) in (1c) or the implied meaning of *not always* (∼*sometimes*) in (2c) are not derived automatically by default, but rather are generated effortfully by canceling the initial literal meaning. In short, the context-driven approach argues that mental effort is required to derive contextual effects to generate scalar implicatures. As a matter of fact, a growing number of recent psycholinguistic studies on native speakers indicate that scalar implicature involves an extra cognitive process evidenced by slower response times in sentence judgment tasks (Bott and Noveck, 2004), longer reading times in self-paced reading tasks (Breheny et al., 2006; Bergen and Grodner, 2012), and delayed eye fixations in a visual world eye-tracking task (Huang and Snedeker, 2009, 2011). For example, Bott and Noveck (2004) examined the generation of SI in adult native speakers of French by measuring response times (RTs) in a sentence-verification task containing underinformative (i.e., pragmatically infelicitous) sentences like (3a). Such underinformative sentences are false with a scalar inference (*some but not all* in (3b)) and true without the inference (*some and possibly all* as in (3c)). Therefore, if participants compute SI (*some but not all*), they would answer ‘False’ to the statement in (3a) because all elephants are mammals. If participants answer ‘True’, it means that participants suspend SI inference and interpret *some* as *some and possibly all* as in (3c).

(3) a. Some elephants are mammals.
  b. ∼ Not all elephants are mammals.
  c. Possibly all elephants are mammals.

Additionally, to investigate the speed of responses, participants in the experiment^1^ were asked to judge such a sentence under two different instructions. Under the ‘Logical’ condition, participants were instructed to interpret *some* as *some and possibly all* whereas under the ‘Pragmatic’ condition, participants were instructed to interpret *some* as *some but not all.*

The results supported the Relevance Theory account. That is, when participants were asked to judge pragmatically, they spent more time in evaluating the underinformative sentences than when they were under the Logical condition. It further indicated that maintaining the SI inference was not effortless in processing and SI computation required extra cognitive effort, as evidenced in longer RTs. This finding was also confirmed by subsequent studies using various methodologies (Degen and Tanenhaus, 2011; Bott et al., 2012).

By employing event-related potentials (ERP) techniques, a large number of studies have investigated the integration of semantic interpretation and pragmatic inference of sentence processing. Noveck and Posada (2003) suggested a smaller N400 effect in underinformative sentences than both semantically and pragmatically acceptable sentences. However, Nieuwland et al. (2010, Experiment 1) reported a similar pattern of N400 in reading underinformative sentences only among participants with low pragmatic ability. By using a picture-sentence verification methodology, Politzer-Ahles et al. (2013) tested Mandarin Chinese speakers’ interpretation of the Chinese scalar item *you de* ‘some of’ in underinformative sentences. The ERP results showed a sustained negativity effect when the pragmatic interpretation of scalar items was not consistent with the context, indicating that suspending pragmatic meaning and activating semantic meaning required extra cognitive effort. More importantly, the authors found a qualitatively different ERP pattern of Chinese scalar items in semantically infelicitous sentences compared to pragmatically infelicitous sentences. It indicates that the reanalysis process of canceling or suspending the pragmatic interpretation is distinctively different from the process of accessing the semantic meaning.

It has been suggested that canceling SI may require additional cognitive efforts (Bill et al., 2015). Being an inference, not linguistically encoded meaning, scalar implicatures can be explicitly canceled without logical contraction. For example, in (4), the inference *not always* of the DSI item *sometimes* is explicitly canceled in Speaker B’s utterance. Similarly, the inference *sometimes* of the ISI item *not always* in (5) is obviously absent in Speaker B’s utterance.

(4) A: Bob was very sick last week. But he sometimes went to school last week.
  B: Yes, in fact, he always went to school last week.

(5) A: Bob was very sick last week. So, he didn’t always go to school last week.
  B: Yes, in fact, he never went to school last week.

There are two routes to the no-inference interpretation. The first route is the following. Under the assumption that a literal meaning without SI is default as proposed by the Relevance Theory, a no-inference reading can be done simply by not generating SI. This way of computing a no-inference interpretation is argued to be cognitively less demanding than generating SI since no-inference is the default interpretation. This is why young children, unlike adults, often prefer literal, no-inference interpretations for scalar items (Smith, 1980; Chierchia et al., 2001; Noveck, 2001; Papafragou and Musolino, 2003). The second route to the no-inference interpretation is to cancel SI after it has been generated first. Whether one’s no-inference interpretation is computed through the first route (i.e., not generating SI at all) or through the second route (i.e., canceling SI) can be teased apart via measuring and comparing response times. We will return to this issue in the methodology section. In this paper, the term *SI suspension* is used generally to refer to the no-inference reading achieved either by not generating SI (in young children’s case) or by canceling SI via re-calculation.

Traditionally, DSIs and ISIs are considered to be the same type of inference; thus, it was assumed that they are involved in the same mechanisms and similar processing efforts. However, recent studies have shown that adults and children behave differently between DSIs and ISIs. One proposal made by Spector (2007) and Chierchia et al. (2012) is that ISIs are obligatory implicatures while DSIs are non-obligatory. According to this proposal, generating DSIs should require more efforts than generating ISIs, but suspending obligatory ISIs should be harder than suspending non-obligatory DSIs. That is, since ISIs are obligatory, interpretations with ISIs (not always ∼ ‘sometimes’) should be easier to process than interpretations without ISIs (not always ∼ never). These two approaches make different predictions about how DSIs and ISIs are generated and processed.^2^

To test whether DSI and ISI are the same kind of inference, Cremers and Chemla (2014) examined the generation of ISI and compared with DSI using a sentence verification task. In their second experiment, participants were asked to judge whether sentences with ISI inference like (6) are true or false against a cover story.

(6) Not all of the [land animals] were fortified.

All sentences were expected to be true under the logical reading (*not all and none*) by suspending the inference but false under the pragmatic reading (*not all but some*). In addition, participants also received explicit instruction on how to interpret these sentences. Half of the participants were assigned to the No-SI group (equivalent to the Logical condition in Bott and Noveck, 2004) and the other half belonged to the SI group (equivalent to the Pragmatic condition). The findings suggested that ISI computation was cognitively more demanding and further indicated a general uniformity for the mechanism that gives rise to both DSI and ISI: scalar implicatures are associated with a delay regardless of the type of SI.

While DSI and ISI seem to be generated in a similar way, their suspension appears to be done through different mechanisms or require varying degree of cognitive efforts as shown in Bill et al. (2016). Instead of using the truth-value judgment paradigm like a sentence verification task, Bill et al. (2016) employed a covered box method developed by Huang et al. (2013). The covered box paradigm differs from the truth-value judgment methodology in that it explicitly offers the non-dominant no-inference interpretation, which encourages participants to consider both inference and no-inference interpretations for the test sentence. That is, while the truth value judgment paradigm is good for examining inference computation, the covered box method is well suited to an investigation of inference suspension.

**FIGURE 1:**
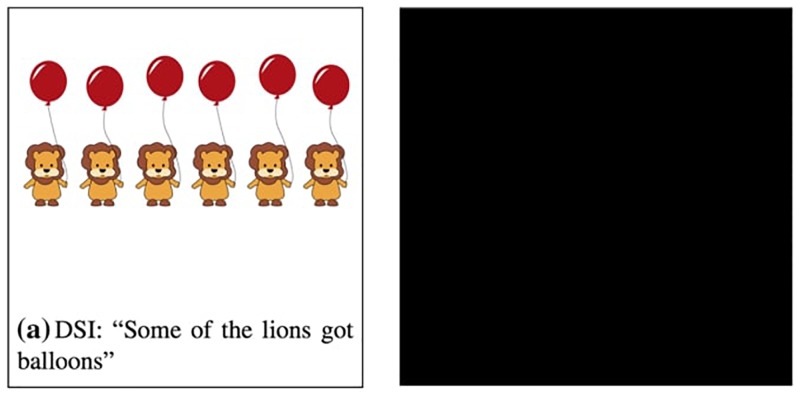
Example trial of the DSI condition [adapted from Bill et al. (2016)].

**FIGURE 2:**
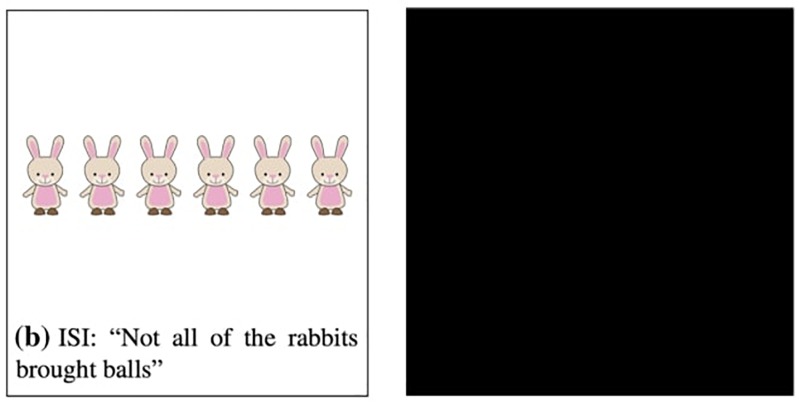
Example trial of the ISI condition [adapted from Bill et al. (2016)].

Using the covered box method, Bill et al. (2016) examined and compared three types of inference: presupposition, DSI and ISI. However, we limit our attention here to Bill et al.’s comparison of DSI and ISI since discussion of presuppositions falls outside the scope of our paper. In Bill et al. (2016), participants were given a test sentence with a visible picture and a black covered box. They were asked to choose the visible picture if it matches the test sentence and choose the covered box if the visible picture does not match the test sentence. Example trials of DSI and ISI conditions are provided in Figure 1, 2, respectively (Figure 1, 2 are adapted from Bill et al., 2016).

The selection of a covered box in each condition indicates the generation of SI whereas the selection of a visible picture suggests the suspension of SI. For instance, in Figure 1, the visible picture depicts a no-inference reading of *some lions*, i.e., *some and possibly all lions*, thus selecting the visible picture indicates suspension of DSI. If participants compute DSI (*some but not all*), they would reject the visible no-inference reading and select the covered box. In Figure 2, the visible picture shows a no-inference reading of *not all*, i.e., *none of the rabbits*. Selecting the visible pictures indicates ISI suspension and choosing the covered-box suggests ISI computation.

There were three groups of English-speaking participants: adults, 4-5 year olds, and 7 year olds. Results showed that adults generated DSI significantly more often than ISI whereas 4-5 year olds and 7 year olds computed ISI significantly more frequently than DSI. Adults were more likely to suspend the inference in ISI than in DSI (a low percentage of selecting covered-box in ISI vs. a high percentage in DSI), while children were more likely to suspend the inference in DSI than in ISI (the opposite percentage pattern to adults).

In sum, there is a general uniformity of processing behavior between DSI and ISI computation such that DSI and ISI are computed at similar rates. However, there are asymmetries between DSI and ISI suspension. English-speaking adults are more likely to suspend ISI than DSI whereas children are more likely to suspend DSI than ISI.

Understanding how DSIs and ISIs are computed and suspended is important not only in linguistic and psycholinguistic theory but also in L2 acquisition theory. Previous research into SI in L2 acquisition has shown that SI computation is not a problem for L2 speakers. In fact, L2 speakers tend to generate SIs more than native speakers do (Lieberman, 2009; Slabakova, 2010; Miller et al., 2016; Snape and Hosoi, 2018). Slabakova (2010) hypothesizes that L2 speakers compute SI more than native speakers because SI cancelation may present challenges to L2 speakers. This issue, however, has not been tested empirically. The present study aims to test whether differences between native speakers and L2 speakers lie in SI suspension rather than SI computation using the covered box paradigm (the logic of this method will be discussed in the next section). Moreover, while there is an increasing number of L2 studies on DSI, little research has been done on ISI in L2 acquisition. To fill this gap, this study examines and compares computation and suspension of DSI vs. ISI by focusing on scalar items like *< sometimes, always >*. Thus, findings of this study would advance our understanding of how alternative meanings are considered in the generation or suspension of SI in an L2.

## Scalar Implicatures in Adult L2 Speakers

The experimental work on the inference computation in adult L2 learners is rather limited. The first study is Slabakova’s (2010) study on how L1-Korean L2-English learners process scalar expressions, such as quantifiers *some* and *all* in their L1 Korean vs. L2 English. The critical experimental item on *some* is (7), which is logically true but pragmatically infelicitous. If participants reject such sentences, it provides clear evidence that participants are able to derive SI and compute the pragmatic reading of *some* as *some but not all*. Acceptance of these sentences indicates that participants suspend SI and generate the logical meaning of *some* as *some and possibly all.*

(7) Some elephants have trunks.
(Slabakova, 2010, p. 2452)

The results showed that Korean learners of English successfully acquired scalar implicatures in their L2. However, differences in response patterns still existed between native speakers and learners. That is, L1-Korean learners of L2-English were more likely than monolingual English or Korean speakers to reject pragmatically infelicitous sentences like (7). One possible explanation proposed by Slabakova (2010) is that it is easier to conjure up situations to make underinformative sentences plausible. For example, if one can think of a situation where some elephants’ trunks got cut due to accidents, the sentence in (7) is felicitous. Another possibility is differential ability to SI suspension. That is, if one cancels the [not all] implicature, the statement in (7) should be interpreted as ‘At least one and possibly all elephants have trunks’, which is true. Since SI suspension arguably requires more cognitive efforts, it might be more difficult to do in an L2 under the assumption that less cognitive resources are available for L2 processing than L1 processing (Green, 1986, 1998)^3^.

A similar study was carried out on L1-English L2-Spanish learners’ interpretation of Spanish quantifiers (Miller et al., 2016) and potential L1 influence in this domain. Unlike Korean that has only one lexical item roughly equal to the English scalar term *some*, Spanish has two: *algunos* and *unos.* While both words have the pragmatic interpretation *some but not all*, only *unos* has the additional logical interpretation *some and possibly all*. With an inherent partitive feature, *algunos* cannot be inferred logically. Imagine a situation that someone has four dogs. When a postman arrives, three out of four dogs barked at the postman in front of the door. In this situation, using either *algunos* or *unos* to mean *some but not all* is felicitous in *Some dogs barked at the postman.* If all the four dogs barked at the postman, the logical interpretation is desired. Thus, it is only felicitous to use *unos*, as in (8a), but infelicitous to use *algunos*, as in (8b).

(8) Context – All four dogs bark at the postman.
  a. *Unos perros ladraron al cartero.*
    “Some dogs barked at the postman.”
  b. *^∗^Algunos perros ladraron al cartero.*
    “Some dogs barked at the postman.”
  (Miller et al., 2016, p. 131)

The fact that Spanish and English do not have a one-to-one mapping on *some* may present further challenges to L2 learners of Spanish. Miller et al. (2016) tested L1-English L2-Spanish learners’ acquisition of the two Spanish scalar terms *algunos* and *unos* through a truth-value video acceptability judgment task. They discovered that English learners were able to obtain a native-like judgment on the two Spanish scalar terms irrespective of the fact that English has a different scalar implicature system. Specifically, not replying on a 1:1 mapping between English and Spanish scalar terms, English learners were less likely to accept *algunos* in non-partitive contexts but were equally likely to accept *unos* despite partitive or non-partitive contexts.

Similar findings were obtained in Snape and Hosoi’s (2018) study on L1-Japanese speakers’ interpretation of *some* in L2 English. The Japanese quantifier *ikutsuka* translates into *some* in English, as in (9).


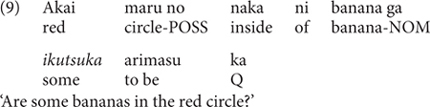


However, unlike English *some* (or Spanish *algunos*), Japanese *ikutsuka* does not have a partitive meaning (*not all*), that is, it does not implicate the *some but not all* meaning. Using a picture-based acceptability judgment task, Snape and Hosoi (2018) examined whether intermediate-level L1-Japanese L2-English learners overaccept pragmatically infelicitous sentences due to L1 transfer and whether such L1 influence would disappear as the proficiency level increases. Conforming to previous studies, Snape and Hosoi (2018) found that L1-Japanese speakers had no difficulty in deriving scalar implicatures despite the mismatches between L2-English *some* and L1-Japanese *ikutsuka* ‘some’. Moreover, there was no proficiency effect.

Lin (2016), employing a series of real-time psycholinguistic experiments on Chinese learners’ acquisition of *some*, contributed to knowledge of L2 speakers’ processing mechanism of scalar implicatures. The first experiment used a Truth Value Judgment task. After reading a context sentence “John has many dictionaries. Some of the dictionaries are used”, participants were asked to judge whether the following target sentences were true or false: “Some and possibly all of the dictionaries are used” or “Some but not all of the dictionaries are used.” Results of the first experiment showed that it was faster for Chinese speakers to compute the pragmatic interpretation of *some* as *some but not all* and it took them more time on rejecting this interpretation. When suspending SI and generating the logical reading (*some and possibly all*), Chinese participants spent almost twice as much time as they did in responding to the pragmatic interpretation. Additionally, they were more likely to reject the logical interpretation. The findings were in line with previous experimental results that adults favor the pragmatic interpretation where the SI inference is present.

The second experiment in Lin (2016) was motivated by the fact that when participants were given unlimited time to respond, they were able to come up with an alternative plausible situation that would fit the sentence at hand. In order to prevent additional brainstorming, in the second experiment, participants were required to respond within a certain amount of time. What is interesting about the finding was when Chinese speakers were pressed for time, the rejection rate of the logical interpretation *some and possibly all* was noticeably increased. In other words, Chinese participants were more likely to reject the suspension of SI when they were under the time pressure. This revealed that suspending scalar items (the logical interpretation) required more cognitive capacity and when L2 speakers’ processing capacity was artificially constrained (e.g., when they were pressed for time), they preferred the cognitively less demanding reading (the pragmatic reading) by computing SI.

In brief, L2 research on SI has shown that generating SI inference is not difficult for L2 speakers and suggests that suspending SI inference may be challenging to L2 speakers. However, the methodology used in previous L2 studies could not tease apart whether differences between L1 and L2 speakers in their rate of SI interpretation is due to difficulties associated with SI suspension in an L2. Our study aims to examine this issue through an investigation of L2 learners’ time course of generating and suspending DSIs and ISIs by employing the covered box paradigm. In this study, we focus on only one type of scalar expressions, namely frequency adverbs like < *never, sometimes, always* >.

## The Present Study

### Research Questions

In light of prior research on DSI and ISI, the present study addresses the following research questions:

RQ1: Do native and L2 speakers differ in *generating* DSI or ISI?
RQ2: Do native and L2 speakers differ in *suspending* DSI or ISI?

### Methodology

**FIGURE 3:**
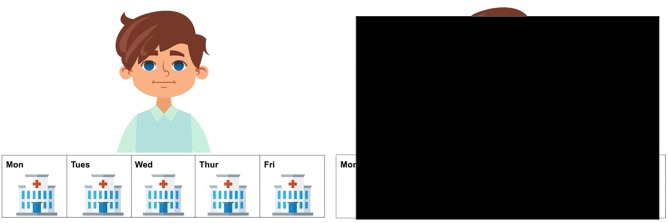
A test trial of the stimulus *Thomas sometimes went to the hospital last week*.

The method used in this experiment was the *covered box paradigm* (Huang et al., 2013), as discussed in Section 2. This paradigm has been successfully applied to explore implicatures (Huang et al., 2013) and presuppositions (Schwarz, 2014; Zehr et al., 2016; Romoli and Schwarz, 2015), especially regarding suspension of an inference. Compared to a traditional picture-selecting task, the difference with the *covered box paradigm* is that it includes a covered box (see the invisible or the hidden picture on the right in Figure 3). Participants were told that there is one picture hidden under the black box. In the current experiment, the instruction on the *covered box paradigm* was if the visible picture matches the stimuli, participants should choose the visible picture. If the visible picture does not match the stimuli, the match must be under the black box and participants should choose the covered box. The advantage of using a covered box is that it is “…useful for testing for the availability of non-dominant interpretations…” (Romoli and Schwarz, 2015, p. 225). The non-dominant interpretation, or the suspension of an inference, is the No-inference visible meaning where the SI inference is absent in the current study. By employing the covered-box paradigm, the SI suspension reading can be displayed explicitly through a visible picture and participants are forced to consider whether the shown picture corresponds to the stimulus. A rejection of the No-inference visible picture (instead choosing the covered box) clearly indicates that the SI suspension or no-inference interpretation is not available to the participants. The same rationale also applies to the dominant interpretation (the Inference visible meaning in the present study). The visible picture in Figure 3 displays a suspension, or No-inference interpretation which is not compatible with an Inference reading that the implicature is present, *Thomas didn’t always go to the hospital.*

#### Test Design

In this experiment, two factors were manipulated in a 2x2 design: SI type and Visible picture. The SI type factor has two levels which are the two kinds of SI we discussed, DSI and ISI. The Visible picture factor has two levels, depending on whether the visible picture shows the SI inference (Inference) or does not display the inference (No-inference). These two factors were crossed to create four conditions: (i) DSI with a visible picture depicting the inference in (10b), (ii) DSI with a visible picture depicting a no-inference reading, like (10c), (iii) ISI with a visible picture depicting the inference in (11b), and (iv) ISI with a visible picture depicting a no-inference reading, as in (11c).

(10) a. DSI: Thomas sometimes went to school last week.
  b. Inference: ∼ Thomas didn’t always go to school last week.
  c. No-inference: Thomas always went to school last week.

(11) a. ISI: Thomas didn’t always go to school last week.
  b. Inference: ∼ Thomas sometimes went to school last week.
  c. No-inference: Thomas never went to school last week.

To convert (10b-c) and (11b-c) into visual stimuli to fit the *covered box paradigm*, the 5-day calendar-strip design was adapted which has been commonly used commonly to investigate the availability of presupposition interpretations was adapted for our study (Schwarz, 2014; Bill et al., 2015; Romoli and Schwarz, 2015; Bacovcin et al., 2016). In this experiment, the calendar-strip contains icons of various activities and locations from Monday to Friday^4^. A continuous appearance of an activity or a location means that this action has been repeated everyday whereas a mixture of activities or locations indicates that the first action has been stopped at some point and a new action has started^5^. Table 1 displays four sample visible pictures for the four target conditions.^6^ The two Inference pictures (12–13) were consistent with a SI interpretation, as in (10b) and (11b). The two No-inference pictures (14-15) illustrated (10c) and (11c) where the icon of hospital in (14) and circus in (15) was shown from Monday to Friday, blocking the SI interpretation. Half of the visible pictures of DSI and ISI were in the Inference condition and were predicted to be selected by both native and L2 speakers, given the preference of the inference or pragmatic interpretation of scalar items in the literature. The other half of the visible pictures were in the No-inference condition and, based on suspension or computation of SI, different response behavior was predicted. Selecting the No-inference visible picture indicates suspension of the SI inference whereas rejecting the No-inference visible picture (instead selecting the covered box) suggests the computation of SI.

**Table 1:** Four test conditions in a 2x2 factorial design: SI type (DSI vs. ISI) and Visible picture (Inference vs. No-inference).

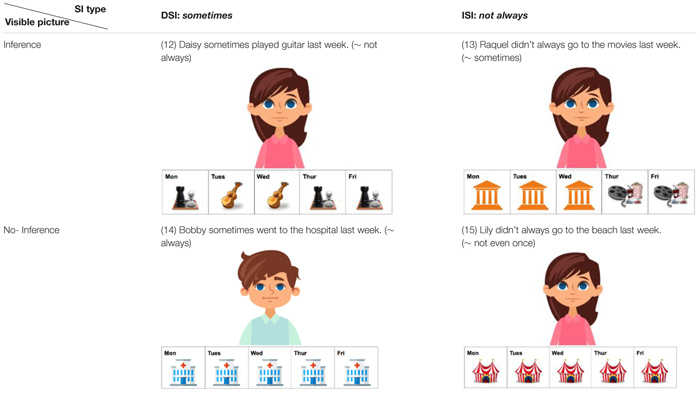

*For ease of exposition, appropriate SI interpretations for each condition are added here in parentheses but they did not appear in the actual experiment.*

In addition to target conditions, we also included controls and fillers, using the same *covered box* method. Half of the visible pictures of controls and fillers matched the stimuli and the other half did not, calling for the selection of the covered box. Controls were used to check if participants understood the task correctly and the sentence stimuli were simple negated and affirmative sentences. For instance, in Table 2, the visible picture of *Louis went to the train station on Wednesday and Friday* had a train station icon on Wednesday and Friday and thus triggered the visible picture selection. The visible picture for *Edward didn’t go to the movies on Thursday and Friday* had a movie icon on Thursday and Friday and participants were expected to choose the covered box.

**Table 2:** Examples of visible pictures for control items.

Simple affirmative sentence	Simple negated sentence
Louis went to the train station on Wednesday and Friday.	Edward didn’t go to the movies on Thursday and Friday.
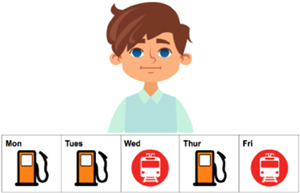	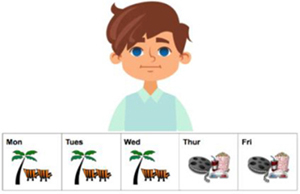


Two types of fillers were included in this experiment. The first type was created using a presupposition trigger *stop* in both affirmative and negated sentences, e.g., *Thomas stopped going to the hospital on Wednesday* and *Bob didn’t stop going to school on Wednesday.* The second type of fillers had *again*, such as *Phoebe went to the gym again on Wednesday during the week.*

#### Procedure and Participants

Twenty-six native English speakers and twenty-four L1-Chinese L2-English learners participated in this study and they were students at a Midwest University in the United States. After signing consent forms^7^, all participants finished three tasks: a brief background questionnaire, a proficiency test and a covered-box task. The background questionnaire collected participants’ information about gender, age and years of studying English. The proficiency test was based on the Common European Framework of Reference for Languages (CEFR) containing 40 items with a maximum score of 40. The summary of participants’ information is shown in Table 3.

**Table 3:** Participants’ background information and proficiency scores.

	Age at testing		Years studying English		Proficiency score	
			
	M (SD)	Range	M (SD)	Range	M (SD)	Range
Native English (*n* = 26)	22.8 (5.55)	19-39	n/a	n/a	39 (1.08)	37-40
High intermediate to advanced^1^ Chinese (*n* = 24)	24.2 (4.34)	18-32	14.2 (2.70)	9-18	35.04 (2.37)	30-39


*^*1*^The proficiency test and the categorization of advanced and intermediate learners were adopted from Cho (2017) where learners with scores above 34 were considered to be advanced learners and those who scored between 26 and 33 belonged to the intermediate level.*

All participants completed the covered-box task on a computer where the program E-prime was used to display stimuli and collect data. The choice of pictures was achieved by clicking on the selected picture via a mouse. A fixation cross for 1000ms was presented at the center of the screen before the display of every stimulus sentence.

Prior to the experimental trials, first, participants finished an icon recognition task which was used to make sure that participants understood the icons correctly. Secondly, participants completed six practice items using the covered-box paradigm to familiarize themselves to the task. Regarding the experimental trials, each participant finished a total of 52 items (16 targets, 16 controls, and 20 fillers) for about 15 min.

### Data Analysis

For the purpose of the analysis, the percentage of selecting covered or visible pictures and response times (RTs) were the two dependent variables in the study. Responses were coded regarding whether the visible or the covered picture was selected. RTs were calculated as the time taken to select a picture. The data were trimmed in two steps. First, participants who selected pictures which obviously did not match the test sentences were planned to be removed, but this did not result in removing any data. The data were further trimmed at +/- 3 standard deviations (SDs) or more from the mean subject RTs. The trimming of extreme data points resulted in the loss of 2.6% of trials in each analysis for L1-Chinese L2-English learners and 2.4% of trials in each analysis for English speakers.

The percentage of selecting visible picture or covered box was analyzed using a generalized logistic mixed-effects regression model. The model had *Percentage* as the dependent variable, *SI type* (2 levels: DSI and ISI) and *Group* (2 levels: Native and L2) as fixed effects, participants and items as random factors.

To correct the skewed distribution of the data, RTs were log transformed and analyzed using linear mixed-effects regression model with *log-transformed RTs* as the dependent variable, *SI type* (2 levels: DSI and ISI) and *Group* (2 levels: Native and L2) as fixed effects, participants and items as random factors^8^.

## Results^9^

### Percentage of Picture Selection

To recapitulate the logic of the covered box method, if participants computed SI, they were expected to choose the visible picture when it depicted the inference and to choose the covered box when the visible picture illustrated no inference. Conversely, if the participant suspended SI, they were expected to choose the visible picture when it portrayed no inference.

When the visible picture showed an inference (as (12-13) in Table 1), both groups selected the visible picture 100 % of the time in the DSI condition and over 97% of the time in the ISI condition. This indicates that both native and L2 speaker groups computed DSI and ISI without any difficulties.

**FIGURE 4:**
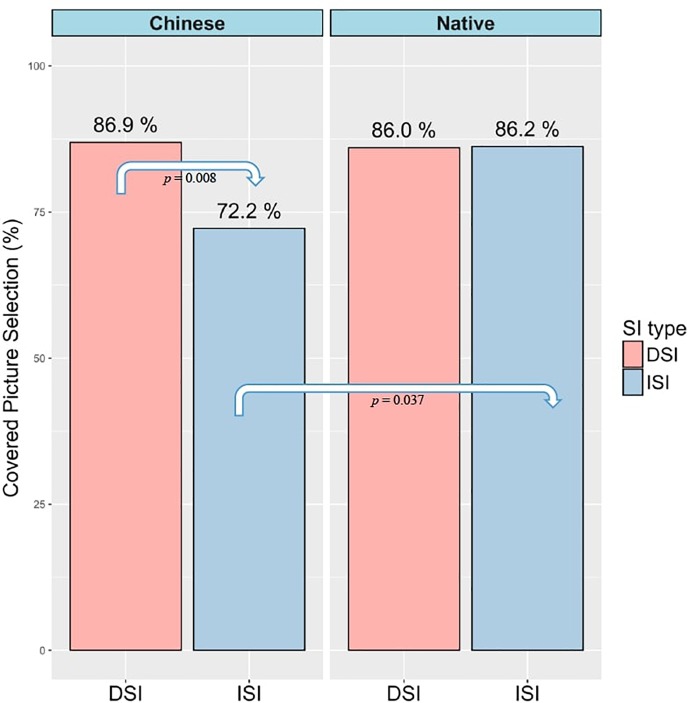
Covered box selection percentage in the No-inference condition of DSI vs. ISI in Chinese and English groups.

The percentage of selecting the covered box in the No-inference condition in DSI and ISI for both groups is visualized in Figure 4. When the visible picture showed an image of no-inference (as (3-4) in Table 1), both native and L2 groups behaved similarly by selecting the covered box about 86% of the time in the DSI condition. There was no significant difference between the two groups (*z* = 0.106, *p* = 0.916). However, the two groups differed in the ISI condition. While native speakers chose the covered box 86.2% of the time, L2 speakers selected the covered box only 72.2%, as visualized in Figure 4. It further suggested that in the No-inference condition of ISI, Chinese speakers were more likely to choose the visible picture than English speakers (Chinese: 27.8% vs. English: 13.8%).

Results from a generalized logistic mixed-effects model suggested a main effect of *SI type* (β = 1.42, *SE* = 0.53, *z* = 2.65, *p* = 0.008) and an interaction between *SI type* and *Group* (β = -1.57, *SE* = 0.74, *z* = -2.11, *p* = 0.035). Post-hoc comparisons indicated that the percentage of covered box selection in the No-inference condition of ISI between Chinese and English speakers was significantly different (*z* = 2.082, *p* = 0.037), as well as the percentage of Chinese speakers between DSI and ISI (*z* = -2.65, *p* = 0.008).

What stood out from the results was the higher percentage of selecting the visible picture in No-inference condition of the ISI by Chinese speakers. The visible picture in this condition represented a logical no-inference interpretation where the SI was suspended. The L2 group was significantly more likely to select the visible picture than the native speaker group in this condition. This could be interpreted in two ways. As noted in the introduction, SI suspension can be achieved through two routes: no computation of SI at all or cancelation of the SI that was initially computed. First, this could mean that L2 speakers, compared to native speakers, have more difficulties in computing ISI. Secondly, this could mean L2 speakers are better at canceling ISI. We will return to this issue in Discussion.

### Response Times (RTs)

Bill et al. (2018) suggested that a comprehensive RT analysis requires the comparative examination between visible picture and covered box selection, in particular when the two types of SI are compared. The reason is that the prediction of RTs is not that RTs will be the same or different between DSI vs. ISI in that we compare two substantially different scalar items, i.e., one with negation and one without negation. Rather, the prediction is whether the overall RT patterns that are categorized by SI computation (choosing the visible picture in the Inference condition and the covered box in the No-inference condition) and SI cancelation (choosing the visible picture in the No-inference condition) are similar or different. Thus, in this study we analyze and compare RTs for selecting the visible picture and RTs for selecting the covered box. Native speakers and L2 speakers’ RTs are summarized in Tables 4, 5, respectively.

**Table 4:** Mean RTs (in ms) for selecting the visible picture vs. covered box by condition (native group).

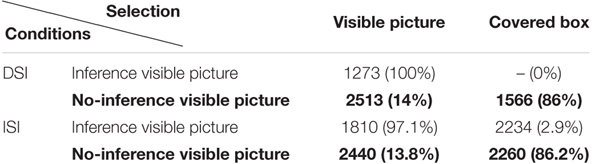

*percentages of selecting visible or covered picture are in parentheses.*

**Table 5:** Mean RTs (in ms) for selecting the visible picture vs. covered box by condition (L2 group).

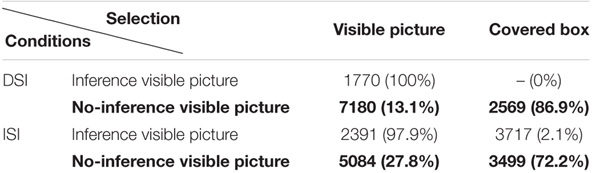

*percentages of selecting visible or covered picture are in parentheses.*

**FIGURE 5:**
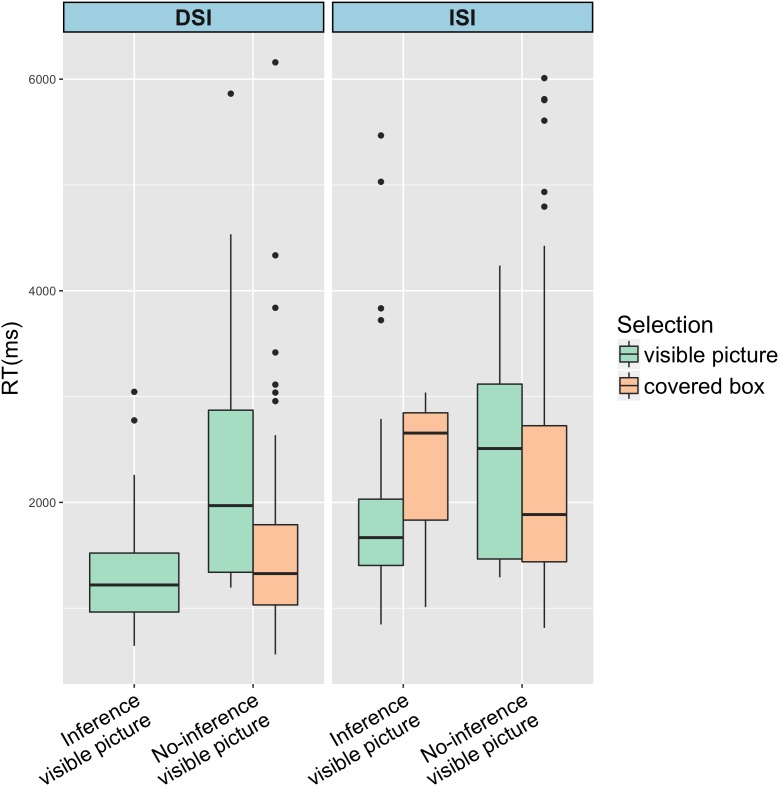
RTs of selecting covered or visible pictures in DSI and ISI by native speakers.

**FIGURE 6:**
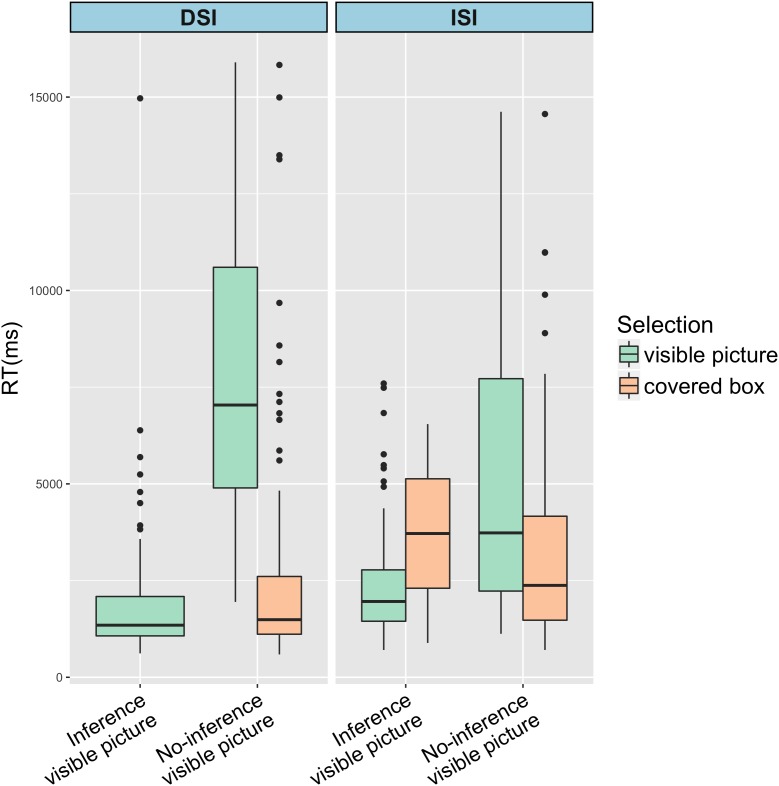
RTs of selecting covered or visible pictures in DSI and ISI by L2 speakers.

As shown in Tables 4, 5, the mean RTs for the covered box selection in the DSI-Inference condition is 0 for both native and L2 speakers since no one selected the covered box in this condition. Tables 4, 5 are further visualized into Figure 5, 6, respectively, by using the *ggplot2* package (Wickham, 2016).

Selecting the visible picture in the Inference condition of DSI was fast for both groups (English: 1273ms vs. Chinese: 1770ms). In the Inference condition of ISI, selecting the visible picture was faster than selecting the covered box for both groups. These results are not surprising since visible pictures in the Inference condition of DSI and ISI were compatible with the reading that SI inference was present. What is more interesting is the RTs in the No-inference condition (in bold in Tables 4, 5) since RTs of visible picture selection represents the time to suspend SI whereas RTs of covered box selection represents the time to compute SI. It seems that both native speakers and L2 speakers were faster in selecting the covered box (computing SI) than the visible picture (suspending SI) in both DSI and ISI^10^.

To investigate RTs of computing SI statistically, log-transformed RTs of the covered box selection in the No-inference condition were fitted for a linear mixed-effects regression model. Type III tests of fixed effects reported significant main effects of *SI type* (*F*(1, 184.50) = 17.142, *p* < 0.001) and *Group* (*F*(1, 44.89) = 10.12, *p* = 0.002) without significant interaction effects between the two factors. It reflected that RTs of selecting the covered box in the No-inference-visible condition of ISI were significantly longer than those of DSI (β = 0.14, *SE* = 0.042, *t* = 3.288, *p* = 0.001) and RTs of English speakers were significantly shorter than Chinese speakers (β = -0.15, *SE* = 0.053, *t* = -2.863, *p* = 0.006). It is not surprising that native speakers were faster than the L2 group. Post-hoc comparisons suggested that it took longer to select the covered box in the No-inference condition of ISI than of DSI for both groups (English: *t* = -3.44, *p* < 0.001; Chinese: *t* = -3.28, *p* = 0.001). In other words, it took longer to compute ISI than DSI for both groups when the non-dominant alternative (no-inference) meaning was explicitly offered.

Another linear mixed-effect regression model was constructed to explore RTs of suspending SI, i.e., RTs of selecting the visible picture in the No-inference condition of DSI and ISI. Type III tests of fixed effects reported significant main effects of *SI type* (*F*(1, 53.474) = 5.22, *p* = 0.026) and *Group* (*F*(1, 23.916) = 14.079, *p* < 0.001) with a marginally significant interaction between the two factors (*F*(1, 55.579) = 3.439, *p* = 0.069). It indicated that RTs of selecting the visible picture in the No-inference condition of ISI were significantly faster than that of DSI (β = -0.29, *SE* = 0.09, *t* = -3.118, *p* = 0.003). RTs of English speakers were significantly shorter than Chinese speakers (β = -0.489, *SE* = 0.121, *t* = -4.031, *p* = 0.0002). Post-hoc comparisons revealed that Chinese speakers were significantly faster in selecting the visible picture in ISI than in DSI (*t* = 3.118, *p* = 0.003) whereas English speakers’ RTs did not contrast significantly between ISI and DSI (*t* = 0.338, *p* = 0.736). It means that unlike native speakers who did not show RT differences in suspending DSI vs. ISI, Chinese speakers were significantly faster to suspend ISI than DSI. The next section moves onto the discussion of these findings.

## Discussion

This study aimed to investigate computation and suspension of two types of SI in L2 acquisition. In this section, we discuss results of the experiment by revisiting the research questions formulated in Section 3.1.

### RQ1: Do Native and L2 Speakers Differ in Generating DSI or ISI?

By employing the covered box method, the ability of generating the SI inference was indicated by participants’ selection of the visible picture in the Inference condition (the inference was present in the visible picture) and the selection of the covered box in the No-inference condition (the inference was absent in the visible picture). For DSI, both groups selected the visible picture 100% when the visible picture showed the inference and preferred the covered box when the visible picture did not show the inference (English 86% and Chinese 86.9%). Moreover, RTs of selecting the visible picture in the Inference condition and the covered box in the No-inference condition revealed that the SI inference was rapidly available to both native and L2 speakers (English: visible picture 1273ms, covered box 1566 ms; Chinese: visible picture 1770 ms, covered box 2569 ms). There was no difference between the two groups in DSI computation. This seems to be in line with findings from previous studies on L2 speakers’ DSI computation (Slabakova, 2010; Miller et al., 2016; Snape and Hosoi, 2018).

As for ISI, both groups selected the visible picture above 97% when the visible picture showed the inference and preferred the covered box when the visible picture did not show the inference (English: 86.2% and Chinese: 72.2%). It is interesting that English speakers were more likely to select the covered box than Chinese speakers when the visible picture showed no-inference and this difference was statistically significant (*z* = 2.082, *p* = 0.037). This seems to suggest that compared to native speakers, it is difficult for L2 speakers to compute ISI when the alternative meaning (no-inference reading in this case) is explicitly offered. In terms of response times, and similar to DSI outcomes, both groups quickly gained access to the SI inference in the Inference condition (English 1810 ms vs. Chinese 2391 ms) and the No-inference condition (English: 2260 ms vs. Chinese: 3499 ms). In short, while L2 speakers computed DSI at nativelike levels, they did not compute ISI as frequently as native speakers.

### RQ2: Do Native and L2 Speakers Differ in Suspending DSI or ISI?

The ability to suspend the SI inference was suggested by the selection of the visible picture in the No-inference condition where the visible picture showed a No-inference reading.

For DSI, both groups selected the visible picture in the No-inference condition at a similar percentage (English 14% and Chinese 13.1%). However, it took significantly longer for Chinese speakers to select the visible picture in the No-inference condition (Chinese 7180ms vs. English 2513ms; *t* = 4.031, *p* = 0.0002). Since the visible picture selection percentages are similar in both native and L2 groups, RT differences between the two groups seem to be a mere quantitative difference. That is, L2 speakers are simply slower than native speakers in suspending DSI.

As for ISI, the two groups differed in the selection of the visible picture. Chinese speakers selected the visible picture at 27.8% whereas English speakers selected at 13.8%. This difference was significant (*z* = 2.082, *p* = 0.037). RT analysis also showed a difference between the two groups of selecting a visible picture (Chinese 5084 ms vs. English 2440 ms; *t* = 1.989, *p* = 0.054). Unlike the quantitative RT differences in suspending DSI, the RT differences between Chinese and English speakers in suspending ISI are qualitative, indicated by the fact that L2 speakers opted for interpretation lacking ISI more than did native speakers.

Taken together, the two types of SI inference were rapidly available to both native and L2 speakers suggested by the quick acceptance of the visible picture that was compatible with an inference reading. It also should be noted that when the visible picture displayed a No-inference reading, the rejection of the visible picture (thus selection of the covered box) was rapid as well for both groups and both types of SI. It further indicated that participants’ preference of an inference reading of SI did not depend on the display of the visible picture (Inference vs. No-inference). Generating SI was overall preferred by both native and L2 speakers. The situation where we observed a significant slow-down for L2 speakers was during selection of the visible picture in the No-inference condition. In this situation, L2 speakers were faced with pressure of opposing alternatives when there was a conflict between the general preference of an inference reading and the visible No-inference reading. What is more interesting is that the pressure of the conflict seemed to be more outstanding for L2 speakers in DSI than in ISI since RTs of selecting the visible picture was significantly longer in DSI than in ISI (DSI 7180ms vs. ISI 5084ms). More importantly, since acceptance of visible pictures in the No-inference condition represents SI suspension, L2 speakers seemed to be able to ‘suspend’ ISI faster than DSI. Another asymmetrical behavior by L2 speakers was that L2 speakers did not compute ISI as frequently as native speakers in that L2 speakers’ percentage of selecting the covered box in the No-inference condition was lower than native speakers (Chinese 72.2% vs. English 86.2%; *z* = 2.082, *p* = 0.037). According to the design of the coved box method, selecting the visible picture in the No-inference condition indicates the suspension of SI. However, as we mentioned in the introduction, there are two substantially different routes that lead to the same behavior (suspending an implicature) and we will discuss the two routes in detail in the following paragraphs. We propose that, in fact, Chinese speakers did not truly suspend the ISI inference because they did not generate the inference in the first place, suggested by their short RTs in selecting the visible No-inference picture of ISI. Instead, they simply selected the interpretation that was visibly offered at hand (the visible No-inference picture).

In sum, comparing DSI and ISI, L2 speakers differ from native speakers in interpreting sentences containing ISI items but not DSI. While L2 speakers did not compute ISI as frequently as native speakers, they ‘suspended’ ISI more frequently and faster than native speakers. These asymmetries between DSI and ISI observed among L2 speakers but not among native speakers pose the following two questions. First, why does ISI computation present more challenges to L2 speakers than DSI? And secondly, why and how do L2 speakers ‘suspend’ ISI more frequently and faster than DSI?

As for the first question, recall the two approaches to DSI vs. ISI discussed in the introduction: the traditional view that treats DSI and ISI as the same type of implicature and the ISI as obligatory implicature (Spector, 2007; Chierchia et al., 2012). According to the traditional view, there should not be any asymmetries between DSI and ISI in their generation and suspension. While our native speaker data seem to support this view, the L2 speaker data clearly suggest that DSI and ISI do not belong to the same group of implicature. Our L2 data cannot be explained within the ISI as obligatory approach either. If DSIs are non-obligatory and ISIs are obligatory implicatures, ISIs should be computed faster and more frequently than DSIs. And DSIs should be suspended more frequently than ISIs. L2 speakers in our study showed the opposite patterns. That is, they computed DSIs more often than ISIs and suspended ISIs more often than DSIs.

To account for our results, we would like to consider differences between DSIs and ISIs in terms of the number of alternative meanings involved. Let us think about structural differences between DSI and ISI. Sentences containing a weaker term that triggers DSI as in (1a), repeated here as (16), are affirmative sentences. ISIs are triggered by negating the stronger term, as in (2a), repeated here as (17). ISIs arise in negative sentences.

(16) Bob sometimes went to school (DSI).
(17) Bob didn’t always go to school (ISI).

Within alternative-based approaches to interpretation, negation is one of the linguistic phenomena where alternatives are computed in order to reach the interpretation by the hearer and numerous psycholinguistic studies have provided empirical evidence to support the claim (Fischler et al., 1983; Hasson and Glucksberg, 2006; Kaup et al., 2007; Lüdtke et al., 2008; Dale and Duran, 2011; Tian, 2014; Tian and Breheny, 2016). For example, understanding the utterance “John didn’t buy a car” requires the hearer to compute the alternative, non-negated meaning “John bought a car” first and then negate it. That is, when interpreting (17), the hearer first computes the non-negated meaning “Bob always went to school” and then negates it. The negated sentence “Bob didn’t always go to school” has two alternative meanings: inference (‘Bob sometimes went to school.’) and no-inference (‘Bob possibly never went to school.’). In other words, in interpreting (17), three meanings should be computed: non-negated meaning, the literal meaning of the negated sentence, and the inferred meaning of the negated sentence. The affirmative utterance in (16), on the other hand, evokes only two alternatives: “Bob sometimes went to school” and “Bob possibly always went to school”. Under the assumption that the more alternative meanings are involved in understanding an utterance, the more cognitive effort is required, (17) containing an ISI item should be more difficult to process than (16) containing a DSI item. This could explain why L2 speakers generated ISI less frequently than DSI.

This issue relates to the second question about SI suspension. As discussed in Bill et al. (2015), speakers go through the following steps in interpreting sentences containing scalar items: (1) accessing the no-inference or literal interpretation; (2) generating SI by default; (3) suspending or canceling SI if needed. We briefly mentioned in the Introduction that achieving no-inference interpretation can be done through two ways: (1) not generating SI at all; (2) canceling SI that was previously generated. Given the three steps proposed in Bill et al. (2015), it suggests that speakers who suspend SI via the first way (not generating SI at all) stop at the first step and therefore, they rapidly generate the no-inference interpretation. On the other hand, speakers who suspend SI via the second way (canceling previously computed SI) must, first, have gone through the derivation of the SI inference and then suspend it. Thus, the re-calculation of meaning is cognitively costly and thus takes longer to undergo all the steps. The asymmetry of suspending DSI and ISI observed among L2 speakers in the present study is that it took Chinese speakers significantly longer to suspend DSI than ISI (DSI 7180ms vs. ISI 5084ms; *t* = 3.118, *p* = 0.003). The longer RTs of suspending DSI by Chinese speakers suggested that they were likely to suspend DSI through the second route, i.e., generating SI and then canceling it. In other words, in reading DSI sentences presented with the alternative no-inference meaning in the visible picture, L2 speakers were able to compute the inference and the alternative reading, and then cancel the inference. Shorter RTs of ISI cancelation were due to the suspension through the first route, i.e., not generating SI at all. When the no-inference reading was offered through the visible picture in the ISI condition, it was difficult for L2 speakers to compute all the alternative readings relevant to the target sentence. So, rather than computing alternatives, L2 speakers opted for the interpretation that was visibly offered.

Finally, it is important to bear in mind that our study only examined frequency adverb scalar items; thus, our results may not be generalizable to all DSIs and ISIs. According to Van Tiel et al.’s (2016) proposal on ‘scalar diversity’, not all DSI items behave the same. For example, Van Tiel et al. (2016) tested 50 participants (20 males and 30 females aged 18-67) on a sentence evaluation task using Mechanical Turk. Participants saw a sentence like John says: *She is intelligent* and were asked a question like *Would you conclude from this that, according to John, she is not brilliant*? Results showed that 100 % of the participants derived SI for < cheap, free > and < sometimes, always > (i.e., 50 out of all 50 participants), while only 6 % of the participants (i.e., three out of 50 participants) computed SI for < intelligent, brilliant > (See Van Tiel et al., 2016 and Van Tiel and Schaeken, 2017 for a detailed discussion on factors influencing the rate of SI derivation).

Furthermore, we would like to consider experimental task effects and potential individual differences in interpreting data. Studies on children showed that children’s logical vs. pragmatic responses differ depending on the task type, instruction, training or experimental setting (see Huang and Snedeker, 2009 for discussion on experimental task effects on inference computation in children). The patterns observed in our study may in part be due to extraneous task effects of the covered-box paradigm related to overall cognitive processing. Task effects in pragmatic inference computation suggest inference processing is closely related to cognitive abilities. In fact, recent studies have also shown that there are individual differences in L2 speakers as well as in native speakers in their computation or suspension of inferences and identified working memory as a main factor affecting inference computation (Marty and Chemla, 2013; Marty et al., 2013). Additionally, many studies have split responders into distinct groups, e.g., pragmatic responders and logical responders or responders with high or low pragmatic abilities, since participants do not have the same threshold of informativeness (Noveck and Posada, 2003; Nieuwland et al., 2010; Tavano and Kaiser, 2010). Experimental task effects and individual differences are therefore important issues for future research on pragmatic processing.

## Conclusion

The main goal of the current study was to examine how L2 speakers compute and suspend the two types of SI, DSI and ISI. While native speakers did not compute or suspend differently between DSI and ISI, L2 speakers showed asymmetrical behaviors to DSI and ISI. More specifically, L2 speakers computed DSI more often and faster than ISI, but suspended ISI more frequently and faster than DSI. The asymmetries of the percentage and time to suspend between DSI and ISI further revealed that L2 speakers went through different routes to suspend ISI and DSI, depending on the extent of alternative meanings involved in the suspension. DSI arises in affirmative sentences while ISI arises in negated sentences which evoke computation of more alternative meanings and re-calculation. It is cognitively more demanding to generate multiple alternative meanings, re-evaluate these meanings and eventually cancel one of them.

## Ethics Statement

The protocol (#2018-0330) was approved by the ED/SBS IRB (Education and Social/Behavioral Science Institutional Review Board) at the University of Wisconsin-Madison. All subjects gave written informed consent in accordance with the Declaration of Helsinki.

## Author Contributions

SF and JC contributed to conception and design of the experiment. SF implemented the experiment and oversaw data collection. Both authors handled analysis of the experiments, contributed to manuscript revision and approved the submitted version.

## Conflict of Interest Statement

The authors declare that the research was conducted in the absence of any commercial or financial relationships that could be construed as a potential conflict of interest.

## Footnotes

1 Bott and Noveck (2004) conducted four experiments. In this paper, we limit our discussion to the first experiment.

2 There are other proposals regarding the nature of DSI, ISI and Presupposition. For example, Chemla (2009) and Romoli (2012) argue that ISIs are presuppositions. The ISI as Presupposition approach states that the stronger scalar terms presuppose their weakest competitor. The statement “John always drinks coffee” presupposes that “John sometimes drinks coffee.” Since presuppositions project under negation, “John doesn’t always drink coffee” should also presuppose “John sometimes drinks coffee.” In this approach, “sometimes” is not an implicature but a presupposition of “not always”. Since our study deals only with DSIs and ISIs, we will not consider the ISI as presupposition approach in discussing our data.

3 The assumption that L2 grammars have limited cognitive resources is based on generally accepted conclusions from psycholinguistic research into bilingualism that both languages are active at all times in the mind of bilinguals (Marian and Spivey, 2003; Dijkstra, 2005; Kroll et al., 2006; Kroll et al., 2015) and having two languages in one mind is cognitively costly because suppressing one language during performance of the other requires cognitive effort (see Green, 1986, 1998 for discussion on inhibitory control/linguistic inhibition in bilingual language performance).

4 A reviewer commented that the calendar strip does not include Saturday and Sunday which could leave room for participants to wonder whether the character might do something over the weekend. We provided our participants with the instruction that the calendar shows the character’s activities last week from Monday to Friday. Sat and Sun were not in the scope of consideration. However, we acknowledge that the 5-day calendar might have triggered some participants to have a partitive meaning.

5 Participants were told that the icon for one day represented that the character went to the place only or did that one activity only. For instance, if a hospital icon appears on Monday, it means that the character only went to the hospital on Monday, nowhere else.

6 As pointed out by a reviewer, activities described in test items vary considerably from going to school to playing guitar. While ‘always going to school’ entails ‘going to school every day’, ‘always playing guitar’ may entail ‘playing guitar every day and all day long’, that is, playing guitar every day for 5 minutes would not be described as ‘always playing guitar’. We acknowledge that this methodological issue could possibly influence participants’ interpretation.

7 Participants were all above 18 years old and gave written informed consent.

8 The mixed-effects generalized logistic model and linear model first included proficiency as a (continuous) fixed factor. However, the results indicated that proficiency was an insignificant factor for both models and, therefore, the simpler models without proficiency were refitted.

9 Tables reporting fixed effects parameters appear in Appendix A.

10 The outlier RTs of both groups are quite long, especially for L2 speakers whose outlier RTs are twice as long as native speakers. As a reviewer suggested, this calls for more implicit online measures (e.g., eye tracking, ERPs) since they would provide more insight on the real-time processing behavior of SI and the integration of semantic and pragmatic meanings.

## Appendix

### Fixed Effects Parameters for Generalized Logistic Mixed-Effects Model and Linear Mixed-Effects Model

**Table 6:** Fixed effects Estimates and Standard Errors for generalized logistic mixed-effects model of percentage of selecting covered or visible pictures.

	Estimate	Std. Error	*z* value	*p* value
SI type (DSI)	1.42	0.53	2.650	0.008**
Group (English)	-0.09	0.81	-0.11	0.916
SI type : Group	-1.57	0.74	-2.11	0.035*

**Table 7:** Fixed effects Estimates and Standard Errors for linear mixed-effects model of selecting the covered box in the No-inference condition for DSI & ISI.

	Estimate	Std. Error	*Df*	*t* value	*p* value
SI type (DSI)	0.14	0.042	258.2	3.288	0.001**
Group (English)	-0.15	0.053	64.0	-2.863	0.006**
SI type : Group	-0.004	0.048	255.2	-0.103	0.918

**Table 8:** Fixed effects Estimates and Standard Errors for linear mixed-effects model of selecting the visible picture in the No-inference condition for DSI & ISI.

	Estimate	Std. Error	*Df*	*t* value	*p* value
SI type (DSI)	-0.29	0.09	58.01	-3.118	0.003**
Group (English)	-0.489	0.121	43.09	-4.031	0.0002***
SI type : Group	0.259	0.139	55.58	1.854	0.069
